# Fasciola Hepatica Infection as a Cause of Severe Hypereosinophilia

**DOI:** 10.4274/tjh.2012.0016

**Published:** 2013-03-05

**Authors:** Meriç Kaymak Cihan, Cahit Babür, Lale Olcay

**Affiliations:** 1 Dr. Abdurrahman Yurtaslan Ankara Oncology Training and Research Hospital Department of Pediatric Hematology, Ankara, Turkey; 2 Ankara Refik Saydam Hygiene Institute, Directorate of Epidemics Research Parasitological Laboratory, Ankara, Turkey

**To the Editor**

Eosinophilia is considered as severe at levels of >5 x 10^9^/L [1]. In childhood, the causes of severe eosinophilia are parasitic infections (visceral larva migrans, trichinosis, hookworm diseases, ascariasis, strongyloidiasis, fascioliasis), allergic disorders, malignant diseases (eosinophilic leukemia, Hodgkin disease, hypereosinophilic syndromes), and some collagen tissue diseases [2].

A boy from a rural area of Turkey, aged 6 years and 10 months, was admitted to our clinic with abdominal pain and severe hypereosinophilia. He had lately developed abdominal pain at the umbilical area, waxing and waning in character. His physical examination was unremarkable.

His laboratory tests are presented in Table 1. 

Since no blast was found in the peripheral blood smear of the patient, secondary causes of hypereosinophilia were investigated. Multiple tests for parasitic infections and collagen tissue disorders were performed (Table 1). Tests for Echinococcus granulosus indirect hemagglutination (IHA) (1/320; normal: <1/160) and Fasciola hepatica IHA (1/5120; normal: <1/160) were positive. A hypodense lesion of 6 x 4 cm was found in the left lobe of the liver upon computed tomography and ultrasonography. Until the Echinococcus granulosus-specific IgE test was revealed to be negative, the patient was given 2 doses of albendazole at 15 mg/kg/day. After 2 weeks, IHA tests for Fasciola hepatica and Ehinococcus granulosus were repeated, which were again positive at 1/5120 (normal: <1/160) and 1/320 (normal: 1/160), respectively. 

The mildly high Echinococcus granulosus IHA was considered to be a cross-reaction with Fasciola hepatica. The parents reported that they had been consuming spring water. For treatment of Fasciola hepatica infection, the patient was given 2 doses of triclabendazole at 10 mg/kg/dose, 1 week apart. The eosinophil counts 1 and 4 months following the second dose declined to 0.64 x 10^9^/L (7.8%) and 0.468 x 10^9^/L (4%), respectively, with no symptoms.

Fasciola hepatica, a liver fluke, is observed in areas of sheep farming and is common in developing countries [3]. More than 180 million people are at risk of Fasciola hepatica infection and 2.4 million people are already infected with this parasite [4]. Eosinophilia is encountered in 14%-82% of patients and may wax and wane during the chronic stage [5,6]. 

In fascioliasis, liver lesions may be present and bile ducts may be observed as thickened and dilated in tomography [7,8]. Fasciola hepatica eggs in stool are generally observed in the acute phase but not in the chronic phase [6]. Serological tests including enzyme-linked immunosorbent assay (ELISA),IHA, complement-fixation, immunofluorescence, counter electrophoresis, and double diffusion are in use. However, all of these sensitive methods may still yield cross-reaction in parasitic infections like Echinococcus [7]. 

For treatment of Fasciola, 2 doses of triclabendazole, at 10 mg/kg/dose 1 week apart, are recommended [6]. 

Fascioliasis develops by the taking in of metacercarial cysts through consumption of aquatic plants (watercress in particular), or of contaminated water like in our case [6]. Fascioliasis can be prevented by taking hygienic precautions. Prognosis is excellent with appropriate treatment. 

Long-duration and repetitive antigen exposure to T lymphocytes by parasites gives rise to excess production of interleukin (IL)-5, IL-4, and IL-13 by the T cells, which stimulates proliferation and survival of eosinophils [9]. 

Our case highlights that in severe eosinophilia, fascioliasis must also be considered, particularly when patients are from rural sheep-breeding areas and drink spring water. 

**Conflict of Interest Statement**

The authors of this paper have no conflicts of interest, including specific financial interests, relationships, and/ or affiliations relevant to the subject matter or materials included

## Figures and Tables

**Table 1:**
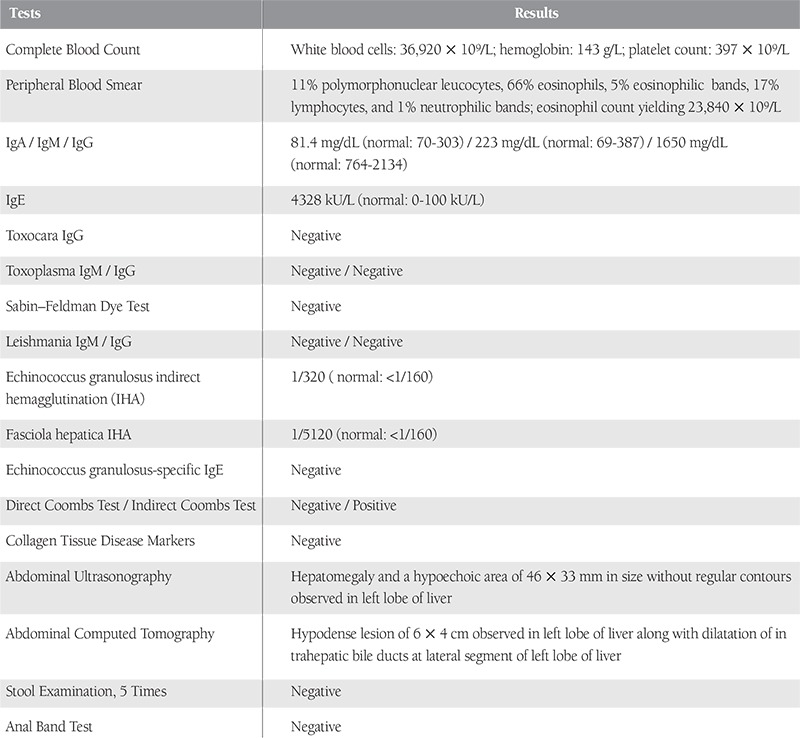
Laboratory test results of the patient.
